# Process Optimization of *Tinospora cordifolia* Extract-Loaded Water in Oil Nanoemulsion Developed by Ultrasound-Assisted Homogenization

**DOI:** 10.3390/molecules29081797

**Published:** 2024-04-16

**Authors:** Varisha Anjum, Uday Bagale, Ammar Kadi, Artem Malinin, Irina Potoroko, Amal H. Alharbi, Doaa Sami Khafaga, Marawa AlMetwally, Al-Seyday T. Qenawy, Areefa Anjum, Faraat Ali

**Affiliations:** 1 Department of Food and Biotechnology, South Ural State University, 454080 Chelyabinsk, Russia; udaybagale@gmail.com (U.B.); artemmalinin3@gmail.com (A.M.); irina_potoroko@mail.ru (I.P.); 2 Department of Computer Sciences, College of Computer and Information Sciences, Princess Nourah Bint Abdulrahman University, P.O. Box 84428, Riyadh 11671, Saudi Arabia; ahalharbi@pnu.edu.sa (A.H.A.); dskhafga@pnu.edu.sa (D.S.K.); 3 Intelligent Systems and Machine Learning Lab, Shenzhen 518000, China; m.metwally@chinamail.com (M.A.); s.qenawy@asia.com (A.-S.T.Q.); 4 Department of Ilmul Advia, School of Unani Medical Education and Research, Jamia Hamdard, New Delhi 110062, India; ariareefa@gmail.com; 5 Department of Analytical Chemistry, Faculty of Pharmacy in Hradec Králové, Charles University, Akademika Heyrovského 1203, 50005 Hradec Králové, Czech Republic; frhtl6@gmail.com

**Keywords:** *Tinospora cordifolia*, nanoemulsion, quality by design, encapsulation efficiency, free fatty acid, response surface methodology (RSM), sonication, ultrasound-assisted nanoemulsification

## Abstract

Nanoemulsions are gaining interest in a variety of products as a means of integrating easily degradable bioactive compounds, preserving them from oxidation, and increasing their bioavailability. However, preparing stable emulsion compositions with the desired characteristics is a difficult task. The aim of this study was to encapsulate the *Tinospora cordifolia* aqueous extract (TCAE) into a water in oil (W/O) nanoemulsion and identify its critical process and formulation variables, like oil (27–29.4 mL), the surfactant concentration (0.6–3 mL), and sonication amplitude (40% to 100%), using response surface methodology (RSM). The responses of this formulation were studied with an analysis of the particle size (PS), free fatty acids (FFAs), and encapsulation efficiency (EE). In between, we have studied a fishbone diagram that was used to measure risk and preliminary research. The optimized condition for the formation of a stable nanoemulsion using quality by design was surfactant (2.43 mL), oil concentration (27.61 mL), and sonication amplitude (88.6%), providing a PS of 171.62 nm, FFA content of 0.86 meq/kg oil and viscosity of 0.597 Pa.s for the blank sample compared to the enriched TCAE nanoemulsion with a PS of 243.60 nm, FFA content of 0.27 meq/kg oil and viscosity of 0.22 Pa.s. The EE increases with increasing concentrations of TCAE, from 56.88% to 85.45%. The RSM response demonstrated that both composition variables had a considerable impact on the properties of the W/O nanoemulsion. Furthermore, after the storage time, the enriched TCAE nanoemulsion showed better stability over the blank nanoemulsion, specially the FFAs, and the blank increased from 0.142 to 1.22 meq/kg oil, while TCAE showed 0.266 to 0.82 meq/kg.

## 1. Introduction

For generations, the highly versatile vine *T. cordifolia*, commonly known as guduchi and a member of the Menispermaceae family, has been utilized in traditional Indian medicine. It is categorized as a Rasayana in the Vedic system, which is a medicine recommended to promote longevity, strengthen the body’s resistance to stress, and operate as an adaptogen [1,2,3]. Numerous articles were published on pharmacological characters, chemical composition, and the validation of therapeutic claims [4,5,6]. The public’s interest in herbal therapy has increasingly declined due to the increasing use of the allopathic medical system and its quick healing effects. About 70–80% of people still utilize herbal treatments as their primary source of health care since they are less harmful and more compatible with the human body [1,3,6]. Due to their relatively minimal half-lives and low bioavailability profile, several herbal bioactives have several health advantages but limited therapeutic potential. Hydrophilic and lipophilic molecules are the two types of compounds generated from plants. Poor oil membrane absorption slows down the biological effectiveness and pharmacokinetics of highly hydrophilic bioactives.

Nanotechnology is characterized by a multidisciplinary approach that comprises the creation and utilization of diverse systems with nanometric dimensions. Nanoemulsions (NE) are lipid-based formulations characterized by droplet sizes in the nanoscale range. These formulations have attracted significant interest as potential drug delivery systems for lipophilic medicines, which exhibit low water solubility. The predictable size distribution, high drug loading, and stability in severe biological environments contribute to the enhanced solubility, penetration across biological membranes, and therapeutic effectiveness of lipophilic pharmaceuticals. Numerous industries, including the food industry, cosmetics, pharmaceuticals, and insecticides, use nanoformulations. Since they are sensitive bioactive ingredient delivery methods, they have recently gained increasing interest in the food industry. However, it is challenging to produce such systems because of their tendency to become unstable during storage [7]. The enchancement of nanoemulsions has been developed using the ultrasound technique. Ultrasound has emerged as a prominent sonoprocess technique owing to its cost-effectiveness, minimal energy demands, user-friendly operation, and enhanced formulation variable control [7,8,9].

Antioxidants, especially polyphenols, are often added to emulsions in the food, cosmetics, and pharmaceutical sectors [10,11,12]. Plant polyphenols have biological properties such as antibacterial, anti-inflammatory, cardioprotective, anticancer, and anti-aging effects. They are frequently used to prevent lipid peroxidation induced rancidity in processed foods and cosmetics. Numerous studies on gallic acid have demonstrated that people can absorb it better than other polyphenols [13]. However, the use of antioxidants in functional food production is restricted because of how quickly they degrade in aqueous solutions. The size of the particles in the dispersed phase has a big impact on how long an emulsion formulation lasts in storage because emulsions with smaller particles are more resilient to gravity separation [14,15,16]. The compounds used modify the nanoemulsion’s stability of coalescence, interfacial tension, rheology, and adsorption kinetics, all of which are associated with the size of the forming droplets [16]. Kumar et al. in 2014 [17] reported that the *T. cordifolia* leaf stem has been incorporated into nanoemulsions for improved therapeutic efficiency.

The process of developing pharmaceutical formulations is intricate and involves several processes and formulation attributes that may have greater impacts on the quality of end product. The interactions and effects of these individual components may influence the CQA (critical quality attribute). One can use the design space to establish a connection between the critical quality attributes and the process inputs [18]. Therefore, through a risk examination by an Ishikawa diagram, screening design for risk analysis, and its optimization by design of experiment (DOE) using the Box–Behnken Design (BBD) of the CQA of the end product, quality by design (QbD) helps to understand the effect of critical processing conditions (CPPs), as reported by Kumar et al. in 2014 [17,19].

In the current research, as per our previous unpublished paper, we use TC stem to extract bioactive compounds (alkaloids and phenolic compounds) under aqueous conditions. This TCAS was incorporated into a nanoemulsion to attain the ideal physical emulsion qualities (particle size, polydispersity index, free fatty acid content, and viscosity). As a result, the QbD approach ensures greater variable control, performance consistency, and product quality. We used the risk assessment matrix (RAM) approach and the Ishikawa diagram to isolate and identify critical attributes according to priority [20]. The QbD process has mostly superseded the one factor at a time (OFAT) approach for drug development, screening, and analysis. This novel approach combines screening with design standardization, yields high-quality results with fewer trial trials, and effectively illustrates the effects of many input variables and their interactions [21]. RSM (response surface methodology) was chosen for this purpose because it combines mathematical and empirical approaches to create models and optimize process parameters in the presence of complex interactions [22,23]. We used polynomial functionality for multivariate optimization. The ideal formulation for the W/O nanoemulsions under investigation was found using BBD. 

The relationship of the bioactive compounds to the emulsifier and surfactant was one of the compositional parameters (PGPR and Tween 20). Sunflower oil was chosen as the continued phase of the nanoemulsion due to its high nutritional content and high oxidation stability; its free fatty acid content and the presence of some micronutrients have been shown to have positive impacts on human health [24]. Maximizing emulsion stability and decreasing particle size with the least amount of emulsifier was the main goal to increase the loading of bioactive compounds (phenolics and alkaloids) [25].

## 2. Results and Discussion

The incorporation of poly-bioactive components into a prepared W/O nanoemulsion is a complicated procedure. Thus, for the successful creation of stable nanoemulsion products, optimization of all major formulation parameters is essential. Prior research has demonstrated the strong dependence of nanoemulsion characteristics on the emulsifying agent and the presence of small surface-active components in either the oil phase or aqueous phase [26]. In the current study, the effects of Amp, oil, and surfactant on the particle size (PS), polydispersity index (PDI), free fatty acids (FFAs), and viscosity were studied using BBD after a careful examination by PBD and a fishbone diagram. 

### 2.1. Solid–Lipid (Oil) Screening

The maximum dissolution of TCAE was observed in sunflower oil. Sunflower oil was therefore chosen as the oil phase to be developed in the formulation.

### 2.2. Initial Risk Assessment by a Fishbone Diagram

**Figure 1:**
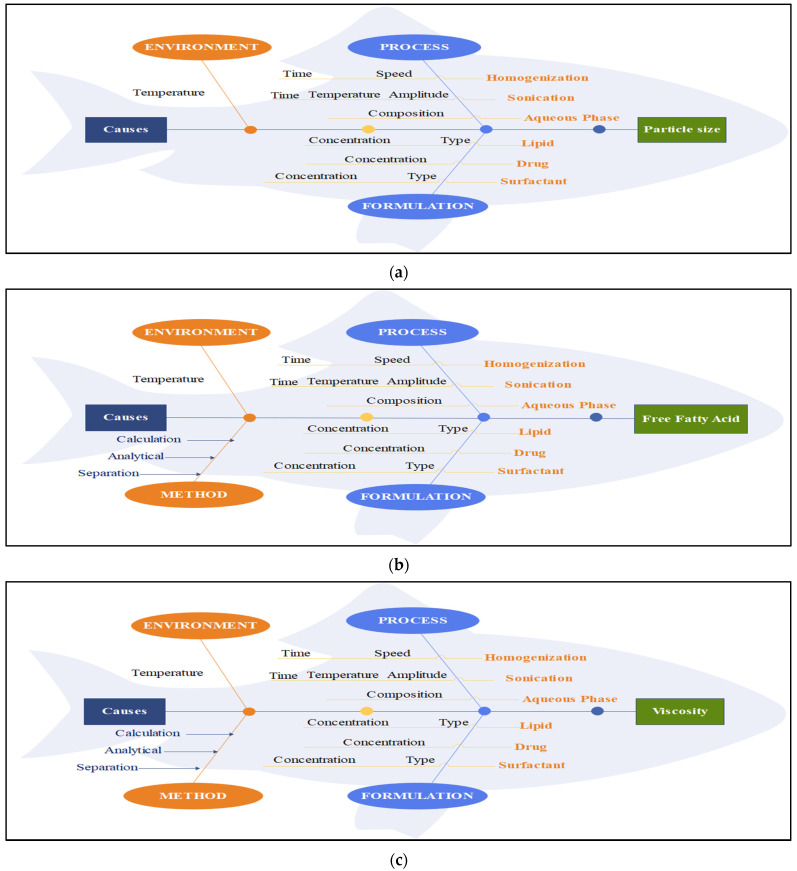
Ishikawa diagrams for (**a**) the particle size, (**b**) free fatty acids, and (**c**) viscosity.

The fishbone diagram helped in studying factors distressing the QAs of nanoemulsion; hence, they were divided into three groups, viz., process, formulation, and nature. All the critical elements were discovered, and their impacts on QAs were assessed by the study, as shown in Figure 1a, Figure 1b, and Figure 1c, respectively. This diagram illustrates that all response parameters, such as PS, viscosity, and FFAs, are impacted by process, method, and formulation constraints.

### 2.3. Primary Identification of Sensitive Variables 

We thoroughly explored the effects of various formulation and process variables. Unlike FFAs and viscosity, the homogenization speed significantly influenced PS. As the homogenization speed increased, droplet flow increased, resulting in a reduction in PS because the high shear rate created at a quicker speed increased the oil droplet viscosity, affecting the emulsification process. High speed, combined with time, is an important aspect of the nanoemulsion dispersion. We discovered that the homogenization time had a significant effect on the PS. As the homogenization duration increased from 1.0 min to 10 min, the PS gradually dropped from 687 nm to 121.50 nm. This is because longer times can lead to colloidal particle instability because of the high energy input that causes colloidal particles to aggregate into larger particles. According to Singh et al. [26], it is not necessary to always have PS decrease as ST increases.

The crucial stage in reducing PS was ST, which had a significant impact on PS. We observed a steady decrease in PS as the ST increased from 1.0 to 15 min. This could be related to the energy that the sonication provided, which caused the coarse dispersion to shrink into nanodroplets, in agreement with Komaiko et al. [27] in their work during the formation of nanoemulsions. For up to 10 min, ST had no discernible effect on viscosity. However, viscosity was shown to decrease at a 15 min ST, which was attributed to drug loss due to particle breakage and decreasing viscosity at a greater ST. There is a direct relationship between the sonication strength and amplitude. The use of a low sonication intensity and low amplitude affects the density and viscosity of the dispersion, which in turn determines its effectiveness. Additionally, the formulation temperature also has an influence on these properties.

Maintaining a temperature above the oil’s melting point is necessary to speed up the dispersion’s disintegration [27]. According to the data, PS was greater than 200 nm at 40 to 60% Amp, proving that ineffective sonication at low intensities could not effectively lower PS. PS dropped to 160 nm when the amplitude was raised from 60 to 80%. PS was unaffected by additional Amp increases. The viscosity was not significantly altered as the Amp was changed. In contrast, the oil content was the primary factor affecting both PS and viscosity. With an increase in oil concentration from 1.0 to 5.0%, PS rose to 342.50 nm. An increase in oil quantity causes an increase in oil droplet viscosity, which in turn affects the homogenizer’s shearing effectiveness in the first stages of emulsification. Additionally, this led to more particle collisions, which accelerated aggregation and raised PS [28]. Sarheed et al. reported that most coconut oil-containing oleic acid nanoemulsions had the largest particle size. The greatest solubility of lidocaine in oleic acid could account for this. As lidocaine was shown to be highly soluble in oleic acid, increasing the amount of the medication solubilized in the oil phase and hence the size of the droplets increases [28]. An alternative explanation could be that viscous dispersion distributes the sonication energy less efficiently than the viscous dispersion does. As the amount of oil increased, the viscosity reached 95%. Increasing oil phase lipophilicity can reduce drug release from the inner phase due to enhanced drug solubility, potentially hindering drug partitioning and release from the emulsion system, Demisli et al. reported that a lipophilic compound nanoemulsion shows increasing viscosity due to the oil phase concentration [29]. The amount of surfactant significantly influences the preparation of nanoemulsions. As the surfactant content increased from 2.0% to 10%, PS progressively decreased from 650 nm to 180 nm. Upon increasing the addition of surfactant from 10% to 15%, PS was not altered considerably. Lower surfactant concentrations can cause the nanodroplets to agglomerate, increasing the PS since there is insufficient surfactant present to cover them. A higher surfactant concentration means that there will be more surfactant available to lower interfacial tension between the two phases, facilitate the oil’s effective emulsification in the aqueous phase, stabilize the nanodroplets, and stop them from coalescing [30]. Research demonstrated that an increase in the surfactant concentration from 2.0% to 10% led to an increase in viscosity, but further increases resulted in a decrease in viscosity. 

### 2.4. Examination of Critical Variables Using PBD and Its Influence 

PBD aids in the initial stages of formulation development by helping to segregate different factors for their influence on the main properties of the W/O nanoemulsion. PBD does not account for factor interaction effects, but it does enable the identification of significant factors with a low run count (12 runs).

**Figure 2:**
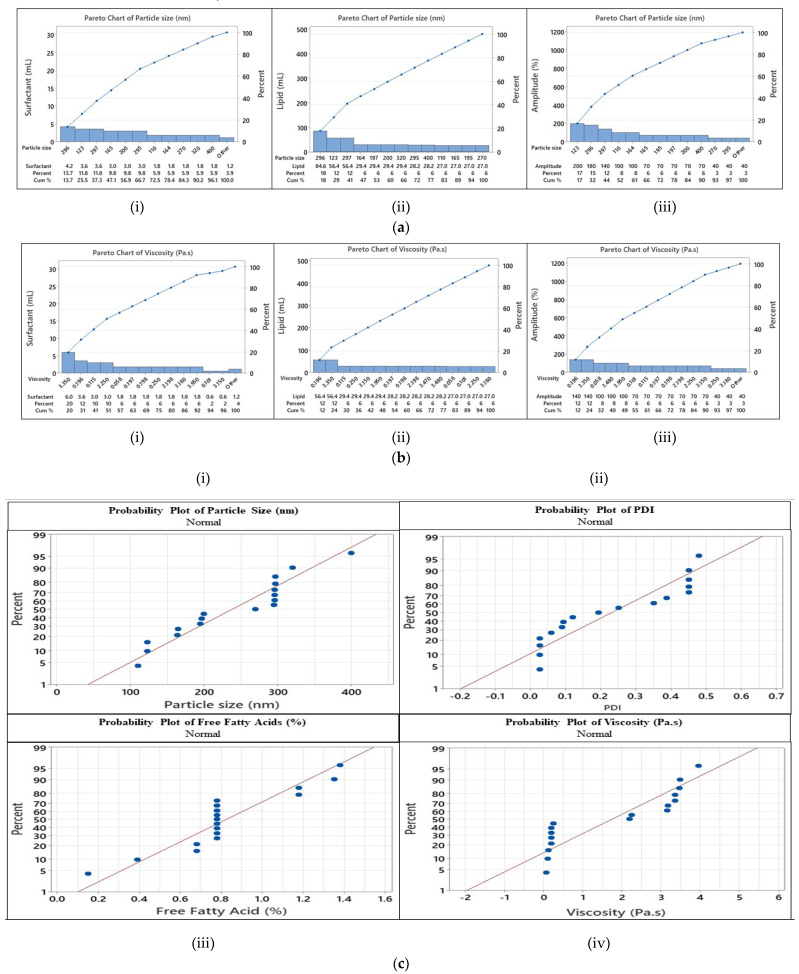
(**a**) Pareto charts of particle size vs. (i) surfactant (ii) oil, and (iii) amplitude. (**b**) Pareto charts of viscosity vs. (i) surfactant, (ii) oil, and (iii) amplitude. (**c**) Probability charts of (i) Particle size, (ii) PDI, (iii) free fatty acids, and (iv) viscosity.

PBD helps in the first stages of formulation development by separating different components according to how they affect the primary nanoemulsion factors. A Pareto chart can be used to rank the most important variables out of all the ones you have chosen. It signifies that impacts that are beyond the reference line are deemed substantial. The Pareto charts in Figure 2a,b show that surfactant, oil concentration, and Amp were considered critical variables for PS and viscosity, along with the probability chart (Figure 2c). 

The normal probability plot revealed that there is a strong association between PS and viscosity, with R^2^ values of 0.97 and 0.96, respectively. According to the results of the Pareto chart and normal plots, it was verified from the effect test parameters that ST, the surfactant concentration, and oil concentration had significant effects with *p*-values ≤ 0.005 for PS, FFA, and viscosity.

### 2.5. Model Fitting Using BBD

We used RSM to examine the effects of independent factors on the viscosity, PS, PDI, and FFA levels of the synthesized nanoemulsion. We used variance and regression analyses to fit the recommended regression models and investigate the statistical significance of the model variables. We assessed statistically significant models and equation terms using the *p*-value index, and found insignificant factors. The results of 17 runs using a BBD are given in Table 1, which includes the experimental design and the corresponding response data.

We used the correlation coefficient (R^2^) to evaluate the quality of the proposed model. We also examined the models’ significance using the F-value and *p*-value. An influence on the related response variable that is more significant would be indicated by a large F-value and a low *p*-value [30,31]. The quadratic regression for the reduced model was in good agreement with the experimental data, since the R^2^ values of PS, PDI, FFA, and viscosity were 0.9889, 0.9876, 0.9914 and 0.9978, respectively, for the W/O nanoemulsions with the addition of enriched TCAE (Table 2). Since R^2^ needs to be ≥ 0.8 to be considered suitably fitted, the resulting R^2^ values show that the models captured the relationship within the selected parameters. Table 2 present the results of fitting models to the data. The ANOVA (analysis of variance) evaluated the significance of the quadratic polynomial models.

The fit response surface models of PS (nm), PDI, FFA (%), viscosity (Pa.s) of the nanoemulsion are given in the equations below.

PS = −28,648.8 − 0.84A + 2043.01B + 1.44C + 5.72AB + 0.006AC + 0.27BC − 40.86A^2^ − 36.18B^2^ − 0.03C^2^ (1)

PDI = 116.89 − 1.05A − 8.42B + 0.054C + 0.34AB − 0.02AC − 0.002BC + 0.04A^2^ + 0.15B^2^ + 0.001C^2^ (2)

FFA = −10.74 − 3.21A + 0.41B − 0.33C + 0.12AB + 0.002AC + 0.01BC − 0.10A^2^ − 0.02B^2^ + 0.0002C^2^ (3)

Viscosity = −151.47 + 22.31A + 13.10B − 1.58C − 0.9AB − 0.0006AC + 0.14BC + 0.81A^2^ − 0.25B^2^ + 0.001C^2^ (4)

#### 2.5.1. Mean Particle Size (PS) of the W/O Nanoemulsion

**Figure 3:**
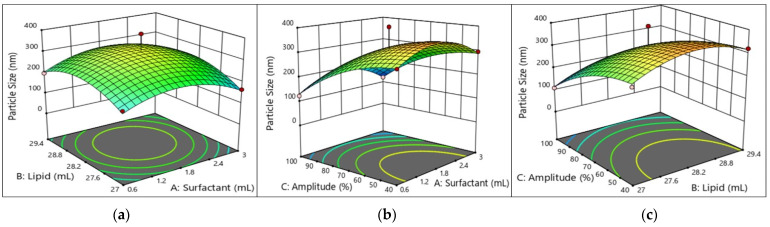
Response surface and contour plots showing effects of (**a**) lipid (oil) and surfactant, (**b**) amplitude and surfactant, and (**c**) amplitude and lipid (oil) on the PS of W/O nanoemulsions.

Several studies have shown that surfactant molecules reduce the interfacial tension between the oil and water droplets by forming an adsorbed layer on the surface of the oil droplets. The lower concentration of the surfactant does not completely cover the droplet surface, but it does lead to droplet coalescence, resulting in increased PS and emulsion instability [28]. Conversely, if the surfactant concentration exceeds a certain limit called the CMC (critical micelle concentration), the continuous phase surface tension reaches the equilibrium position and the droplet size increases again. Orugun et al. reported the same argument when studying a gallic acid nanoemulsion formation, where the surfactant concentration increased while reaching its critical micelle concentration, providing a larger emulsion droplet [32]. We also observe that an increase in the PS occurs when the oil phase ratio or oil concentration is high. More oil moves and builds up below the system when the oil phase ratio is pretty high. This can cause the nanoemulsion to flocculate or coalesce, but a high water phase can make more droplets hit each other and faster. The sonication amplitude decreases the particle size with increasing sonication intensities. As a result of this higher sonication intensity, due to high pressure, strong waves break the insoluble phase and provide a homogeneous solution, resulting in an oil droplet that reduces the particle size. We conclude that the PS of the water in oil-based nanoemulsion is positively affected by the surfactant and oil composition as shown in Figure 3. The major effect on the model developed for the mean PS of the W/O nanoemulsion corresponds to the linear and quadratic terms of surfactant and oil (A^2^, B^2^, *p* < 0.01; C, *p* < 0.001). Equation (1) provides an interaction between AB and BC to correlate the result for the particle size (AB and BC show significant term), and even in Table 3 we can see the lack of fit value for the particle that is non-significant (0.1219), which is fitted to model.

#### 2.5.2. Polydispersity Index (PDI) of the W/O Nanoemulsion

**Figure 4:**
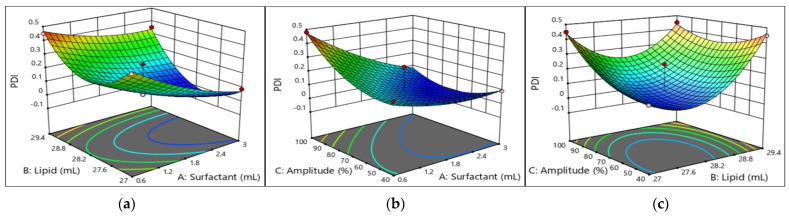
Response surface and contour plots showing effects of (**a**) lipid (oil) and surfactant, (**b**) amplitude and surfactant, and (**c**) amplitude and lipid (oil) on the PDI of W/O nanoemulsions.

The concentration of droplets in various size classes is defined by the PS distribution, which is a characteristic of emulsions. PDI values greater than 0.7 suggest that the sample has an extremely broad particle size distribution and might be inappropriate for analysis using the dynamic light scattering approach, whereas low PDI values indicate a monodisperse nanoemulsion with strong kinetic stability. It was discovered that the oil and surfactant had major impacts on the PDI of W/O nanoemulsions, while SA had a negative impact on the PDI. As oil and surfactant concentrations increase, the PDI also decreases, as it was found that the dynamic of the size of the emulsion decreases as further increases in the surfactant concentrations allow the nanoemulsion show a narrow particle size distribution resulting in higher TCAE absorption. As we have seen in Figure 4, as sonication increases, the PDI also increases, which loses its narrow distribution into a broad distribution as the value goes beyond 0.5. In particular, there was a substantial impact on the PDI from the linear term of surfactant (A, *p* < 0.01) and the linear and quadratic term of oil (B, *p* < 0.05, B^2^, *p* < 0.001). Equation (2) offers an interaction between AB and AC to correlate results for PDI (show significant term), and even in Table 3, we can see a lack of fit value for the particle that was non-significant (0.5471), which is fitted to the model. These values provide a well-fitted mathematical model for the equation.

#### 2.5.3. Free Fatty Acid (FFA) Content of the W/O Nanoemulsion

Given that as little as 0.1% can hasten the synthesis of hexanal in a W/O emulsion, FFAs are trace components present in commercial oils that are potent pro-oxidants. Remarkably, the capacity of FFAs to stimulate oxidation is decreased when their degree of unsaturation increases. Because linear fatty acids can lower the negative charge of the emulsion droplets more than polyunsaturated fatty acids, they may find it easier to reach the interface of the emulsion droplets. As unsaturation increases, FFAs in surfactant micelles counteract the oxidation tendency, leading to an increase in oxidative stability. As the surfactant concentration increases, the FFAs decrease in mean viscosity, reducing the possibility of oil oxidation. It was also discovered that, as predicted, increasing the degree of unsaturation of the FFAs (e.g., linolenic vs. oleic acids) led to higher oxidation rates, which were supported by the unsaturated FFAs’ higher oxidizability. This O/W nanoemulsion made the oil–water interface more negatively charged, which made it easier for prooxidant metal ions to stick to it and helped the oil oxidize. The presence of pro-oxidants, antioxidants, or emulsifiers in the water phase and at the W/O interface, among other factors, significantly affects the oxidative stability of the O/W nanoemulsion, even though oil oxidation in these emulsions may be similar to that in bulk oil (i.e., caused by direct exposure of the lipid phase to air). Numerous research studies have investigated the effect of antioxidant addition on lipid oxidation in W/O emulsions. The oil and Amp had a substantial impact on the FFAs of the W/O nanoemulsions. The model showed that the linear interaction had an impact on FAAs, and the quadratic term of surfactant (A, AB, A^2^, C^2^, *p* < 0.05, BC, *p* < 0.001) suggests the model’s importance.

#### 2.5.4. Viscosity of the W/O Nanoemulsion

The visuals of an emulsion are one of the key factors influencing its overall quality. The size, concentration, and spatial distribution of emulsion droplets determine light scattering and absorption; hence, the composition and structure of the emulsion influence its overall appearance [28]. The viscosity of the current nanoemulsion increased as the oil content increased, the amount of oil dropped in a firm volume increased, and the contact of these droplets led to an alteration in the viscosity (Table 3). TCAE interacts with polar or hydrophilic groups to produce steric repulsion. The side chains of TCAE can expand expressively into the oil phase, and by keeping enough space between them, their steric repulsion can stop the bonds from aggregating. Molecules on the surfaces of water and oil increase both the surface viscosity and perceived viscosity of the oil in the film between droplets. These two effects inhibit aggregation and raise viscosity. Surfactant and Amp had substantial effects on the viscosity of the W/O nanoemulsions. This result could be due to the sonication process breaking down polymer chains. By lowering interactions, this breakdown alters the connections between polymer molecules, decreasing viscosity and bringing the behavior closer to that of Newton. The model’s ability to specifically demonstrate how viscosity was impacted by the linear and quadratic terms of the surfactant ratio (AB, A^2^, *p* < 0.01) and the linear and quadratic terms of the Amp ratio (BC, C^2^, *p* < 0.01) highlights the model’s significance. 

#### 2.5.5. Interpretation of the Response Surface Model and Contour Plot

**Figure 5:**
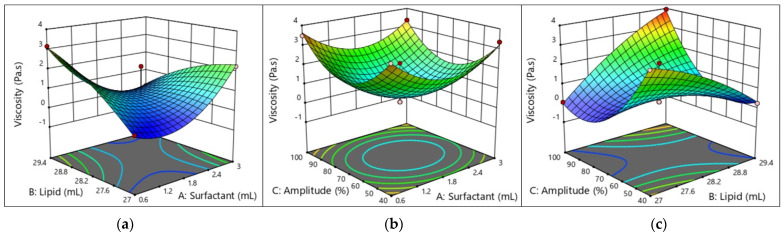
Response surface and contour plots showing effects of (**a**) lipid and surfactant, (**b**) amplitude and surfactant, and (**c**) amplitude and lipid on the viscosity of W/O nanoemulsions.

**Figure 6:**
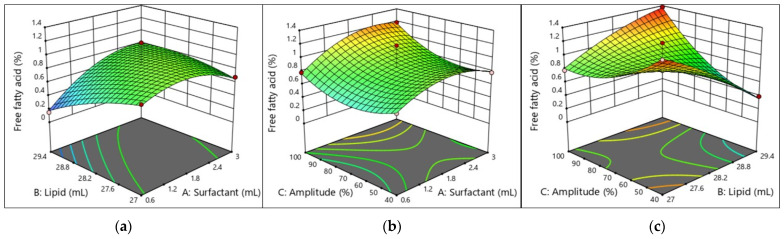
Response surface and contour plots showing effects of (**a**) lipid (oil) and surfactant, (**b**) amplitude and surfactant, and (**c**) amplitude and lipid (oil) on FFAs of W/O nanoemulsions.

The contours of the four response surfaces can be superimposed to create an overlay plot that illustrates the range of ideal conditions and the area where all response surfaces can achieve their optimal values. Figure 3, Figure 4, Figure 5 and Figure 6 show the response surfaces and their respective contour plots, illustrating the effects of the surfactant, oil fraction, and Amp on PS, PDI, viscosity, and FFAs. The lowest values of PS, PDI, viscosity and the highest values of the FFA responses represent the optimal zone of surfactant, oil, and Amp combinations. 

It was evident that high surfactant and oil fractions along with Amp led to an increase in W/O nanoemulsions’ attributes. Other researchers have also noted the latter, concluding that several endogenous bioactive chemicals in sunflower oil have surface-active characteristics [33,34]. Additionally, with an increase in the surfactant ratio, the W/O nanoemulsions’ characteristics improved. However, as the emulsifier ratio increased over 10% *w*/*w*, the characteristics of the W/O nanoemulsions declined. This suggests that the system became unstable due to a rearrangement of the existing colloids generated by a PGPR ratio more than 10% *w*/*w*.

As can be seen in Figure 3, Figure 4, Figure 5 and Figure 6, the addition of PGPR and Amp frequency was beneficial to the nanoemulsion, helping to develop improved emulsion characteristics. This is explained by PGPR’s capacity to reduce the nanoemulsion viscosity. The most favorable features of the W/O nanoemulsion were obtained at an optimal composition of 0.7307 mL of surfactant, 27 mL of oil, and 70% Amp.

#### 2.5.6. Optimization of the W/O Nanoemulsion 

An ideal formulation for the W/O nanoemulsion preparation would have the lowest PS, PDI, and viscosity, as well as the maximum FFA%. Since a target value of 1.0 (on a scale of zero to one) was chosen for attractiveness, the PS was optimized at 204.08 nm, which may be attained by combining 3 mL with 70% Amp. The optimum values of PDI (0.09), FFA% (0.915) and viscosity (1.148) with a desirability of 0.711 can be achieved with the incorporation of 2.43 mL of surfactant, 27.61 mL of sunflower oil, and an amplitude of 88.62%. Additionally, we optimized the W/O nanoemulsion by determining the optimal composition and administering TCAE.

Lastly, duplicate assenting experiments were conducted using the optimal settings for each unique response as a means of validation. Table 3 displays the values that the model’s equations predict in addition to the experimental data. The findings from the desirability optimization analysis and the experimental outcomes did not differ considerably. After that, the model can be utilized to incorporate an enriched TCAE to optimize the W/O nanoemulsion protocol.

### 2.6. Design of Experiment (DOE) and Evaluation Parameters for the Developed Formulation

The preparation of an enriched TCAE nanoemulsion is a complex procedure. Thus, for the successful preparation of stable nanoemulsion products, optimization of all key formulation parameters is essential. In this study, Test 1 (100 mM) and Test 2 (200 mM) of TCAE were calculated with respect to the percent yield as per pharmacopoeia human dose and nanoemulsions were prepared using the optimized parameters, namely, 2.43 mL of surfactant, 27.61 mL of sunflower oil, and an amplitude of 88.62%. Table 4 displays the PS, PDI, FFA, and viscosity values for each bioactive chemical (blank, test medication) in the evaluated W/O nanoemulsion formulations.

**Figure 7:**
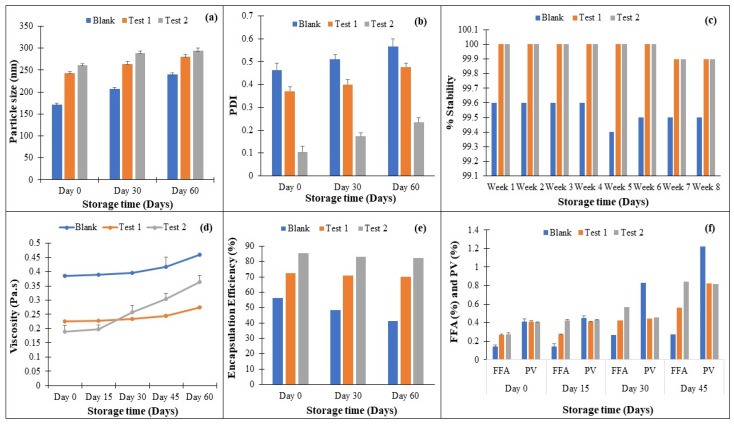
Impact of storage conditions on different properties of the emulsion: (**a**) PS, (**b**) PDI, (**c**) ESI, (**d**) viscosity, (**e**) FFAs and PV, and (**f**) encapsulation efficiency (where Test 1 and Test 2 indicate 100 mM and 200 mM of TCAE, respectively).

Figure 7a,b shows that the PS and PDI of the blank sample vary significantly with respect to the storage time when compared to Tests 1 and 2, which contain TCAE. The values for the fresh sample blank, Test 1, and Test 2 range from 171.9, 243, and 261 nm to 240, 277, and 293 nm after 60 days of storage (*p* < 0.01), respectively. It was discovered that the polymer dispersity of the nanoemulsion remains homogeneous in the TCAE-based emulsion. Droplet size rises over time as it progresses through the continuous phase, increasing the chance of an accident. Emulsions reduce interfacial areas in non-equilibrium systems. Various breakdown processes, including creaming, sedimentation, flocculation, Ostwald ripening, and coalescence, provide free energy. Stability over a 6-week period, where sample tests give higher stability (*p* > 0.05) (Figure 7c) even though they include bioactive compounds, make them suitable as functional food candidates. Figure 7d depicts the effect of storage time on the viscosity of a nanoemulsion sample, and it was discovered that all samples had acceptable stability (ranging from 99.6 to 100%); however, when compared to each other, the encapsulated sample nanoemulsion performed slightly better than blank sample (*p* < 0.01) after 60 days but was non-significant after day 15 (*p* > 0.05), as previously observed since particle size increased over a 6-week period. Figure 7d depicts the impact of the stability time on the viscosity of samples; the blank sample showed substantial variations in viscosity (0.3897–0.46 mPa.s) compared to the test samples (0.225–0.27 mPa.s) during a two-month period. Blank samples have smaller particle sizes, which means increased viscosity, or an increase in the surface area to volume ratio, and this characteristic changes during nanoemulsion stability [34].

Figure 7e depicts the FFAs and PV in relation to one another. The FFA value for all samples is less than 5%, which is within an acceptable range for a healthy fatty acid content. However, test samples may contain slightly more FFAs (*p* < 0.01) after 45 days, whereas the difference from the blank was non-significant after 15 days (*p* > 0.05) because the presence of an antioxidant in the TCAE-encapsulated nanoemulsion reacted with it during initial lipid oxidation to produce polymerized unsaturated fatty acids. Furthermore, the storage FFAs increase with increasing time for the blank sample (0.142–0.277) and the TCAE sample (0.26–0.566). A higher PV, on the other hand, is more susceptible to oil oxidation; blank samples exhibit increased PV during 30 days of storage (*p* < 0.01), ranging from 0.41–1.22, compared to 0.413–0.822 for bioactive emulsion samples (*p* > 0.05), whereas after 45 days, the difference was found to be significant (*p* < 0.01). Natural oils include polyphenols, which are strong antioxidants. Enrichment with these antioxidants in proper concentrations could be a beneficial approach. Antioxidant biocompounds, such as thiols and phenols, can reduce lipid oxidation in W/O emulsions by acting as electron donors. Figure 7f provides the outcomes of the % encapsulation efficiency during a period of 2 months. It was found that the blank sample had a significantly(*p* < 0.01) varying % EE from 56.33 to 41.33 in comparison to the TCAE nanoemulsion (*p* < 0.01), which showed almost unchangeable variation (85.22–82.33%). As we already found that with respect to an increasing concentration of drug, %EE also increased, and we mention that the lower the PS, the greater the loading of the drug.

**Figure 8:**
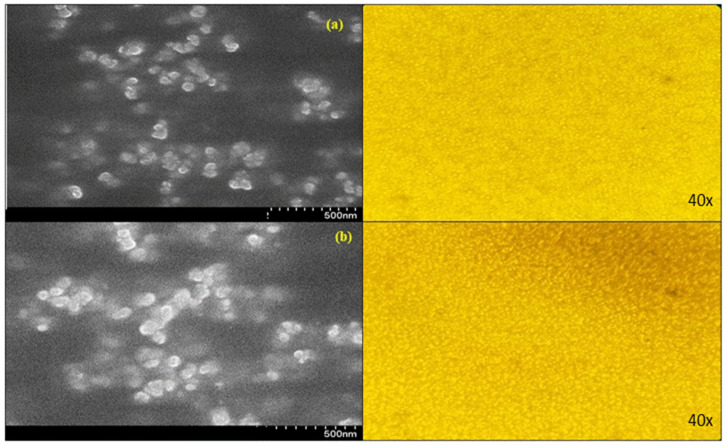
Scanning electron microscopy and optical microscopy images of optimized nanoemulsions for samples containing (**a**) 100 mM and (**b**) 200 mM TCAE.

From the scanning electron microscopy and optical microscopy images of the optimized W/O nanoemulsion, it was observed that best optimized condition is the drug-loaded nanoemulsion as follows: 2.43 mL of surfactant, 27.61 mL of sunflower oil, and an amplitude of 88.62%. Under this condition, we have tested a microscopic analysis using a laser conclave microscope and SEM and found that the droplet of oil is in a spherical shape with a particle size ranging from 315 to 415 nm (Figure 8). As for the case of drug-loaded nanoemulsions, 50% loading shows particles that are less aggregated but have better emulsion stability, whereas 100% drug-loaded nanoemulsions show more aggregated particles.

## 3. Materials and Methods

### 3.1. Materials

Tween 20 (polyoxyethylene-20-sorbitan monolaurate; HLB = 16.70), polyglycerol polyricinoleate (PGPR), gallic acid, and quercetin were obtained from Sigma Eldrich (St. Louis, MO, USA). Sunflower oil was procured from the local market in Russia and it was stored at 10 °C before the experiments. Deionized water was used in the preparation of all nanoemulsions. *T. cordifolia* stem powder was purchased from local vendors in India.

### 3.2. Screening of Solid–Lipid (Oil)

An accurately measured quantity of drug was added in increasing concentrations and mixed in various oils and stirred until it dissolved completely. The solution was diluted with a methanol/chloroform mixture equally and the content of dissolved drug was determined using a UV spectrophotometer (Shimadzu UV-2700, Shimadzu, Kyoto, Japan) at 270 nm [35].

### 3.3. Preparation of the Water in Oil (W/O) Nanoemulsion

As per our previous unpublished work, we extracted alkaloids and phenolic compounds from the aqueous extract of TC. Further, this TCAE was used for encapsulation in the water in oil nanoemulsion. As reported by Polychniatou and Tzia in 2018 [25] with some modifications, the fabrication of the nanoemulsion was carried out. The water phase of the nanoemulsion was formulated by dissolving 0%, 0.1% (100 mM), and 0.2% (200 mM) *w*/*v* of TCAE. Briefly, for 100 mL of emulsion, the W/O emulsion was prepared by mixing 40 mL of water and 0.2% Tween 20 for 5.0 min in a magnetic stirrer. Afterwards, 2.0% of surfactant (PGPR) was added to 60 mL of oil. The aqueous solution was slowly added to the oil solution and gently shaken at 3000 rpm on a magnetic stirrer to obtain a stable emulsion (10 min). Further, the solution was probed for 15 min with 7 s pulse on and 5 s pulse off mode at an amplitude of 70% to keep the temperature below 35 °C [36]. The tested (W/O) nanoemulsion was configured as follows: sunflower oil, PGPR, and amplitude. These configurations were finalized for the optimization using BBD.

### 3.4. Experimental Design

#### 3.4.1. Initial Risk Assessment

Initially, a fishbone diagram was deduced to find out the critical variables that can affects the CQAs of the TCAE nanoemulsion. Particle size (PS), free fatty acids (FFAs), viscosity, and encapsulation efficiency (EE) were short listed as product QAs [37].

#### 3.4.2. Primary Identification of Sensitive Variables 

After determining which factors from the Ishikawa diagram would have an impact on the product QAs, we conducted a preliminary analysis of the variables based on risk priority. We changed one process and formulation variable at a time, keeping the others constant, to perform a preliminary optimization. We evaluated PS, FFAs, viscosity, and EE to determine the ideal lower and higher values for a screening design study.

#### 3.4.3. Plackett–Burman Design (PBD) for Risk Analysis 

The Plackett–Burman design was used to perform an initial screen of important factors based on their respective influence on the PS and EE of the nanoemulsion. We used the results from the preliminary inquiry to determine the high and low values for each factor. We built the PBD using 12 runs of Minitab version 16 (Minitab Inc., State College, PA, USA). The following were important variables: X1—homogenization time; X2—homogenization speed; X3—sonication time (ST); X4—sonication amplitude (Amp); X5—oil concentration; and X6—surfactant concentration. PS, viscosity, and FFAs were the chosen responses based on the transdermal administration of drug-containing nanoemulsions, as minimizing PS and reducing viscosity exhibit quicker release of active components and enhance drug permeation [38].

### 3.5. Ultrasonic Treatment Using the Box–Behnken Design (BBD)

Initially, a 1.0 mg/mL concentration of the aqueous solution of TCAE was prepared with a minor modification. The sonicator probe was introduced into the solution 1.0 cm below the suspension’s top surface. Explorations were conducted for the impact of sonication (ultrasonic) parameters at three coded levels between −1 to +1: concentration of surfactant [0.6 (−1)–3.0 (+1) mL], concentration of oil [27 (−1)–29.40 (+1) mL], and ultrasound input power level [40 (−1)–100 (+1) % of the total input power]. To assess the pure error, five replicates of the center point were included in the experimental design, which had 17 combinations. All the experiments were performed in triplicate.

#### Optimization by RSM

One-way ANOVA was performed after Tukey’s test at *p* < 0.05 levels to examine the homogeneity of variance for all responses and the significant impact of different combinations. The decision was made to enhance the ultrasonic degradation conditions by utilizing 3-factor BBD. Three levels (−1, 0, and +1) of surfactant, oil, and ultrasonic intensity were explored. There were 17 experimental combinations developed by the BBD. The response variable was fitted using the following quadratic model, where Z is the dependent variable. The coded independent variables are Y_1_, Y_2_, and Y_3_. The variable regression coefficients for the model intercept, linear, quadratic, and interaction effects are, in order, b0, bj, bjj, and bjk. Based on the R_2_ determination coefficients, adjusted R_2_ coefficients, predicted R_2_ coefficients, CV%, and appropriate precision values, the suitability of the constructed mathematical models was assessed.

Z = β_0_ + β_1_Y_1_ + β_2_Y_2_ + β_3_Y_3_ + β_11_Y_1_^2^ + β_22_Y_2_^2^ + β_33_Y_3_^2^ + β_12_Y_1_Y_2_ + β_13_Y_1_Y_3_ + β_23_Y_2_Y_3_

### 3.6. Physicochemical Analysis of Nanoemulsions

#### 3.6.1. Determination of the Particle Size (PS) and Polydispersity Index (PDI)

We used an electrochemically built pH meter to determine the pH of the produced nanoemulsions. Using the DLS approach, a Nanotrac FLEX particle size analyzer (Microtrac MRB, Retsch, Haan, Germany) assessed the PS distribution. In the particle size analysis, the nanoemulsions were diluted 1:100 with distilled water to achieve a uniform particle suspension. The PS and PDI were measured using light scattered from the sample, which was put on a Nanotrac external probe.

#### 3.6.2. Nanoemulsion Stability Index (ESI)

The stability index of the nanoemulsion was calculated by the method reported by Bagale et al. [7] with some modifications. The nanoemulsions were centrifuged for 30 min at 5000 rpm after being held in a hot water bath at 80 °C for 30 min. They were moved to an ice bath for 15 min. We calculated the degree of creaming and/or sedimentation by dividing the volumes of cream and sediment production by the total volume of the emulsion samples. The emulsion stability index (ESI%) was calculated with the formula in Equation (5), where HE is the initial emulsion height, HC is the cream layer height, and HS is the sedimentation phase height.

Nanoemulsion stability index (ESI; %) = HE − [(HS + HC)/HE] × 100 (5)

#### 3.6.3. Determination of the Viscosity of the Nanoemulsion

The nanoemulsion viscosity was found using an SV-10 vibration viscometer (A&D Company Ltd., Tokyo, Japan). The SV-10 vibration viscometer is designed to measure the dynamic lightness of various liquid media in real time. The measurement using the viscosity analyzer was carried out in a continuous wide range (0.3–1000 mPa.s) at a temperature of 25 °C. Viscosity measurements were performed for each sample in triplicate with a volume of 45 mL [7].

#### 3.6.4. Determination of the Free Fatty Acids (FFAs; Total Titratable Acidity)

The Ca 5a-40 technique was utilized to calculate the total titratable acidity of each sample [37]. A total of 2.0 g of oil was mixed into 25 mL of an alcoholic–ether solution (1:2 *v*/*v*), followed by the addition of 1.0% phenolphthalein indicator in ethanol. The final solution was titrated using a 0.1 M sodium hydroxide solution until it developed a pink color, persisting for a minimum of 30 s. We calculated the % FFA content by multiplying v by M and dividing the result by 28.20, where % FFA is represented as percent free fatty acids, v is the volume of solvent used, M corresponds to the molarity of the NaOH solution, and m is the molecular weight of the oil sample used. We expressed the results in oleic acid content (% *m*/*m*) and conducted all analyses in triplicate.

#### 3.6.5. Peroxide Value

The peroxide value (PV) in extracted fat was investigated according to AOAC Official guidelines [39] and the findings were expressed as meq/kg fat.

#### 3.6.6. Microscopic Image Analysis and Scanning Electron Microscopy (SEM)

The examination of the nanoemulsion pattern was carried out using a Nikon E-800 (Kawasaki, Japan) bright field light microscope at high magnification (40×). After refrigerating at 4 °C for 24 h, the emulsions were examined. A single water droplet (3 μL) was previously placed on a slide (76 × 26 mm), and following the extension, a coverslip (24 × 32 mm) was placed over the emulsion (4.0 μL). A Nikon DXM-1200 digital camera (Shanghai, China) was used to monitor and pictures were captured. Particle sizes were extracted from the images using the Olympus BioSystems GmbH (Soft Imaging System GmbH, Münster, Germany) image analysis-D 5.0 tool. The analyses were carried out in triplicate. The distribution of PS was calculated for diameters less than 4 μm, between 4 and 7 μm, 7–10 μm, and more than 10 μm. For SEM imaging to obtain the structural morphology of nanoemulsion using a Joel SEM (Jeol Ltd., Tokyo, Japan) instrument, the sample was first sputtered with gold for conductivity and then mounted on a slot for image processing at a magnification of 15,000–20,000 KV [7].

#### 3.6.7. Determination of Encapsulation Efficiency (EE)

The encapsulation of bioactive phytocomponents is characterized by EE. The EE was computed using Chou et al.’s [40] technique with slight changes. The produced nanoemulsion (15 mL) was centrifuged for 30 min at 5000 rpm (5 °C) after passing through the membrane filter. The UV (Shimadzu UV-2700, Japan) absorbance was measured at 417 nm and 520 nm after centrifugation. Triplicates were used for all measures. %EE was calculated according to Equation (6).

%EE = (Total amount of drug added − free drug)/Total amount of drug added × 100(6)

#### 3.6.8. Total Flavonoid and Phenol Determination 

Total phenol and flavonoid concentrations in TCSE were determined using the aluminum chloride and Folin–Ciocalteu (FC) methods, respectively, with slight changes [41]. The total flavonoid and phenolic contents were expressed as microgram (µg) quercetin equivalent/milligram (mg) and microgram (µg) gallic acid equivalent/milligram (mg) of the sample, respectively.

#### 3.6.9. Antioxidant Activity Using DPPH

The antioxidant scavenging property and IC_50_ of TCAE were investigated spectrophotometrically at 515 nm using DPPH against a blank [41]. The inhibition percentage of the DPPH radical was calculated using Equation (7):

Inhibition percentage of the DPPH radical: [(Abs_control_ − Abs_sample_)/Abs_control_] × 100 (7)

#### 3.6.10. Data Analysis

Means and standard deviations (SDs) of all replicates were calculated using GraphPad Prism 8. The results were analyzed using the statistical software Minitab 16 and Design-Expert 13 (State-Ease Inc., Minneapolis, MN, USA) was used to calculate the coefficients and optimizing quadratic and cubic polynomial models. These combinations were tested for various parameters. A significance level of *p* ≤ 0.05 was used for all evaluations.

## 4. Conclusions

The effect of different formulation and process variables, such as PS, FFA, and EE, on CQAs of the aqueous *T. cordifolia* extract nanoemulsion was understood using a QbD approach. The fishbone graphic facilitated the initial risk assessment for the formulation development process. The range of each component for additional research was defined with the aid of a preliminary investigation. With the help of PBD and BBD, we completely understood the major, interactive, and quadratic effects of the many variables in the high-speed homogenization method followed by the ultrasonication method, as well as their respective influences on product quality attributes. The study’s findings suggest that RSM is a useful method for simulating response variable fluctuations in relation to independent factors. Water in sunflower oil nanoemulsions with an added bioactive component were statistically optimized with respect to their compositions. We developed second-order polynomial models to predict the W/O nanoemulsions’ PS, PDI, viscosity, FFAs, PV, EE, and ESI%, and obtained regression equations with significant results. All the responses that were evaluated (PS, PDI, turbidity, FFAs, PV, EE, and ESI%) were represented in the experimental data, and a quadratic model fit them well. The ratio of PGPR (polyglycerol polyricinoleate) to sunflower oil had a substantial impact on the characteristics of the W/O nanoemulsions. More favorable W/O nanoemulsions were produced when the PGPR ratio was raised. However, the emulsion characteristics declined as the PGPR ratio went above 10% *w*/*w*. The anticipated and experimental values did not differ considerably, according to all the data. The results are useful because they help us make sunflower oil-based, optimized W/O nanoemulsions that are stable enough and contain a highly bioactive enhanced extract. Furthermore, the surface tension study’s findings suggest that the *T. cordifolia* stem-enriched extract may play a significant role in influencing the nanoemulsion aqueous phase’s air/water surface tension. The type and nature of the additional bioactive component, as well as its physicochemical characteristics in connection to their affinity for the air/water interfaces, will determine this effect. Future studies of these nanoemulsions will be fortified on dairy or bakery products and their in-depth analyses. As Russia is a country where most people consume dairy or bakery product, such products would be beneficial for health.

## Figures and Tables

**Table 1:** Results obtained for different treatments designed by the RSM methodology.

Run	Independent Variables			Observed Responses			
	Surfactant (mL)	Lipid (Oil; mL)	Amplitude (%)	Particle Size (nm)	PDI	Free Fatty Acids (%)	Viscosity (Pa.s)
1	1.8	28.2	70	400	0.25	1.18	2.198
2	0.6	28.2	100	123	0.48	0.78	3.48
3	3	29.4	70	200	0.35	0.78	0.115
4	1.8	28.2	70	296	0.027	0.78	0.198
5	1.8	27	100	110	0.45	0.78	0.058
6	1.8	28.2	70	297	0.027	0.78	0.197
7	1.8	28.2	70	297	0.027	0.78	0.196
8	1.8	28.2	70	296	0.027	0.78	0.196
9	0.6	29.4	70	197	0.45	0.15	3.15
10	1.8	29.4	40	320	0.45	0.39	0.25
11	0.6	28.2	40	296	0.193	0.68	3.47
12	1.8	29.4	100	164	0.45	1.38	3.95
13	3	28.2	100	123	0.06	1.18	3.35
14	0.6	27	70	195	0.387	0.78	0.101
15	3	28.2	40	295	0.093	0.78	3.35
16	1.8	27	40	270	0.12	1.35	3.18
17	3	27	70	165	0.09	0.68	2.25

**Table 2:** Analysis of variance for the response surface of PS, PDI, FFA, and viscosity of the W/O nanoemulsion.

Average Particle Size ^a^ (PS; nm)						
Source	Sum of Squares	df	Mean Square	F-Value	*p*-Value	Remarks
**Model**	91122.21	9	10,124.69	69.11	<0.0001	Significant
A—Surfactant	98.00	1	98.00	0.6689	0.4404	
B—Lipid	2485.13	1	2485.13	16.96	0.0045	
C—Amplitude	54615.12	1	54,615.12	372.78	<0.0001	
AB	272.25	1	272.25	1.86	0.2150	
AC	0.2500	1	0.2500	0.0017	0.9682	
BC	4.00	1	4.00	0.0273	0.8734	
A^2^	14582.41	1	14,582.41	99.53	<0.0001	
B^2^	11429.09	1	11,429.09	78.01	<0.0001	
C^2^	4338.57	1	4338.57	29.61	0.0010	
**Residual**	1025.55	7	146.51			
Lack of Fit	750.75	3	250.25	3.64	0.1219	not significant
Pure Error	274.80	4	68.70			
**Cor Total**	92147.76	16				
**Polydispersity Index ^b^ (PDI)**						
**Model**	0.5498	9	0.0611	62.13	<0.0001	significant
A—Surfactant	0.1051	1	0.1051	106.91	<0.0001	
B—Lipid	0.0533	1	0.0533	54.21	0.0002	
C—Amplitude	0.0426	1	0.0426	43.36	0.0003	
AB	0.0097	1	0.0097	9.87	0.0163	
AC	0.0256	1	0.0256	26.04	0.014	
BC	0.0272	1	0.0272	27.69	0.0012	
A^2^	0.0143	1	0.0143	14.57	0.0066	
B^2^	0.2025	1	0.2025	206.00	<0.0001	
C^2^	0.0478	1	0.0478	48.64	0.0002	
**Residual**	0.0069	7	0.0010			
Lack of Fit	0.0026	3	0.0009	0.8192	0.5471	not significant
Pure Error	0.0043	4	0.0011			
**Cor Total**	0.5566	16				
**Free Fatty Acids ^c^ (FFA; %)**						
**Model**	1.40	9	0.1556	128.94	<0.0001	significant
A—Surfactant	0.1326	1	0.1326	109.92	<0.0001	
B—Lipid	0.0990	1	0.0990	82.07	<0.0001	
C—Amplitude	0.1058	1	0.1058	87.70	<0.0001	
AB	0.1332	1	0.1332	110.43	<0.0001	
AC	0.0225	1	0.0225	18.65	0.0035	
BC	0.6084	1	0.6084	504.30	<0.0001	
A^2^	0.1054	1	0.1054	87.40	<0.0001	
B^2^	0.0062	1	0.0062	5.11	0.0584	
C^2^	0.2024	1	0.2024	167.77	<0.0001	
**Residual**	0.0084	7	0.0012			
Lack of Fit	0.0045	3	0.0015	1.54	0.3347	not significant
Pure Error	0.0039	4	0.0010			
**Cor Total**	1.41	16				
**Dymanic Viscosity ^d^ (Pa.s)**						
**Model**	42.30	9	4.70	357.34	<0.0001	significant
A—Surfactant	0.1613	1	0.1613	12.27	0.0100	
B—Lipid	0.4399	1	0.4399	33.45	0.0007	
C—Amplitude	0.0432	1	0.0432	3.29	0.1128	
AB	6.72	1	6.72	510.83	<0.0001	
AC	0.0000	1	0.0000	0.0019	0.9664	
BC	11.63	1	11.63	884.64	<0.0001	
A^2^	7.98	1	7.98	606.85	<0.0001	
B^2^	0.1307	1	0.1307	9.94	0.0161	
C^2^	14.14	1	14.14	1074.81	<0.0001	
**Residual**	0.0921	7	0.0132			
Lack of Fit	0.0912	3	0.0304	137.03	0.112	Non-significant
Pure Error	0.0009	4	0.0002			
**Cor Total**	42.39	16				

(**Note:** Each experiment and analysis of samples was performed in triplet) ^a^ The coefficient of determination (R_2_) of the model was 0.9889. ^b^ The coefficient of determination (R_2_) of the model was 0.9879. ^c^ The coefficient of determination (R_2_) of the model was 0.9914. ^d^ The coefficient of determination (R_2_) of the model was 0.9978.

**Table 3:** Experimental and predicted values of the response under optimized conditions.

Response (Unit)	Predicted Value (Mean ± SD)	Experimental Value (Mean ± SD)
Particle size (nm)	181.20 ± 0.97	171.50 ± 0.73
Polydispersity index	0.07 ± 0.14	0.25 ± 0.54
Free fatty acids (%)	0.86 ± 0.73	1.18 ± 0.97
Viscosity (Pa.s)	0.59 ± 0.24	2.20 ± 1.32

**Table 4:** DOE responses of the developed W/O nanoemulsions.

Response	Blank	Test 1 Drug (100 mM Added)	Test 2 Drug (200 mM Added)
Particle size (nm)	171.90 ± 1.43	243.00 ± 1.64	293.66 ± 1.25
Polydispersity index	0.25 ± 0.08	0.37 ± 0.14	0.10 ± 0.06
Free fatty acids (%)	0.86 ± 0.21	0.26 ± 0.08	0.27 ± 0.13
Viscosity (Pa.s)	0.59 ± 0.12	0.22 ± 0.04	0.19 ± 0.06

Test 1 (50% drug as per human dose) and Test 2 (100% drug as per human dose).

## Data Availability

The data presented in this study are available in article.
